# Clinical and diagnostic evaluation of flank pain in female patients: a retrospective cohort study of 3350 patients

**DOI:** 10.1186/s12905-026-04573-1

**Published:** 2026-05-25

**Authors:** Dursun Baba, Ahmet Çalışkan, Ahmet Yıldırım Balık, Ekrem Başaran, Arda Taşkın Taşkıran

**Affiliations:** 1 Department of Urology, Duzce University Faculty of Medicine, Duzce, Türkiye; 2 Department of Urology, Akçakoca State Hospital, Duzce, Türkiye

**Keywords:** Diagnostic evaluation, Imaging, Flank pain, Urolithiasis, Urinary tract infection, Urinary WBC

## Abstract

**Background/Objectives:**

This study aimed to determine the distribution of confirmed urological diagnoses in female patients presenting with flank pain and to evaluate the diagnostic performance of clinical, laboratory, and imaging parameters in identifying urological pathology.

**Methods:**

This retrospective cohort study included 3350 female patients (18–65 years) who presented with flank pain at the urology outpatient clinic of Düzce University Hospital between January 2020 and September 2024. Patients were categorized into two groups based on the presence (Group 1, *n* = 1831) or absence (Group 2, *n* = 1519) of a confirmed urological diagnosis. Serum creatinine, urinary white blood cells (WBCs), urinary red blood cells (RBCs), and clinical and imaging findings were analyzed. Receiver operating characteristic (ROC) analysis and multivariable logistic regression were performed to assess diagnostic performance.

**Results:**

A confirmed urological diagnosis was identified in 54.7% of patients, with urinary tract infection (27.8%) and urolithiasis (19.8%) being the most frequently documented conditions. Urinary WBC levels demonstrated higher diagnostic performance than serum creatinine (AUC: 0.82 vs. 0.76). In multivariable analysis excluding imaging-derived variables, urinary WBC levels (OR: 3.12, 95% CI: 2.64–3.68, *p* = 0.01) and serum creatinine (OR: 2.08, 95% CI: 1.72–2.51, *p* = 0.03) were significantly associated with confirmed urological diagnoses. Combined models incorporating laboratory and clinical parameters showed improved discriminative performance (AUC up to 0.86). Imaging findings, including hydronephrosis and urinary stones, were more frequently observed in patients with confirmed urological diagnoses (*p* = 0.01).

**Conclusions:**

Urological conditions constitute a substantial proportion of flank pain presentations in female patients evaluated in a urology outpatient setting. While laboratory and imaging findings provide useful diagnostic information, no single parameter alone is sufficient for accurate differentiation, and a combined clinical and diagnostic approach is required.

## Introduction

Flank pain is a common yet diagnostically challenging symptom encountered across multiple medical disciplines, including urology, internal medicine, gynecology, orthopedics, and emergency medicine. Its broad differential diagnosis includes both urological and nonurological conditions, making accurate and timely evaluation critical for optimizing patient management [1, 2]. While urological disorders such as urinary tract infections (UTIs), nephrolithiasis, and structural abnormalities are among the most common causes of flank pain, nonurological conditions such as musculoskeletal disorders, gastrointestinal diseases, neurological syndromes, and gynecological pathologies may present with similar symptoms, leading to diagnostic uncertainty [3, 4].

In female patients, the diagnostic approach for flank pain is more complex than that in male patients. The broader range of potential etiologies in women includes gynecological disorders (e.g., ovarian cysts, endometriosis, and pelvic inflammatory disease), gastrointestinal conditions (e.g., irritable bowel syndrome, diverticulitis), and musculoskeletal pain syndromes. This diversity complicates initial clinical evaluation and often necessitates a structured, multidisciplinary diagnostic approach [5, 6]. Pain characteristics provide useful diagnostic clues: acute onset colicky pain is often associated with urological causes such as ureteral obstruction, whereas chronic dull pain is more commonly linked to musculoskeletal disorders [7].

Despite advances in diagnostic imaging and laboratory testing, flank pain remains a significant clinical challenge, leading to frequent emergency department visits and extensive diagnostic workups [8]. Inappropriate or delayed diagnoses can result in unnecessary antibiotic use, redundant imaging studies, and even hospitalizations, increasing healthcare costs and patient anxiety [9]. Identifying reliable diagnostic markers that can differentiate between urological and nonurological causes is therefore crucial.

Epidemiological studies indicate that the prevalence of urinary stone disease in the Turkish adult population is approximately 14.8%, with regional variations [10]. However, a substantial proportion of patients presenting with flank pain do not have an identifiable urological pathology, underscoring the need for a comprehensive evaluation that considers alternative etiologies [11]. Laboratory markers such as serum creatinine and urinary white blood cells (WBCs) are frequently used in clinical practice, yet their diagnostic accuracy in distinguishing urological from nonurological flank pain remains unclear. Additionally, imaging modalities such as ultrasonography (USG) and noncontrast computed tomography (CT-KUB) play crucial roles in the assessment of structural abnormalities, yet their use must be optimized to balance diagnostic yield and healthcare resource utilization [12, 13].

However, most existing studies primarily focus on isolated etiologies or single diagnostic modalities, and data specifically addressing the integrated clinical and diagnostic evaluation of flank pain in female patients remain limited [1, 2, 5]. In particular, evidence from urology outpatient settings—where patients are often preselected based on suspected urological conditions—is scarce. Therefore, rather than solely describing etiological distributions, there is a need for studies that evaluate the combined diagnostic contribution of clinical presentation, laboratory findings, and imaging modalities within a real-world clinical context.

This study aims to retrospectively analyze the distribution of confirmed urological diagnoses in female patients presenting with flank pain to a urology outpatient clinic. Additionally, we evaluated the diagnostic value of key clinical parameters, laboratory markers, and imaging findings to assess their combined performance in identifying urological pathology. By focusing on a structured diagnostic approach within a defined clinical setting, this study seeks to provide clinically applicable insights that may support more efficient and targeted evaluation strategies.

## Materials and methods

### Study design and setting

This study was designed as a retrospective cohort study conducted at the urology outpatient clinic of Düzce University Faculty of Medicine. The study period covered patient admissions between January 2020 and September 2024.

Ethical approval for the study was obtained from the Non-Interventional Clinical Research Ethics Committee of Düzce University (approval number: 2021/230; date: 15 November 2021). The study was carried out in accordance with the principles of the Declaration of Helsinki.

Given the retrospective design of the study and the use of anonymized patient data, the requirement for informed consent was waived by the ethics committee.

### Study setting and population

The study was conducted at the urology outpatient clinic of a tertiary care university hospital. The study population consisted of female patients aged 18–65 years who presented with flank pain during the study period.

A total of 3350 patients who met the eligibility criteria were included in the analysis. All patients underwent clinical evaluation at the time of presentation, and only those with available laboratory and imaging data were considered for inclusion in order to ensure a standardized diagnostic assessment.

As the study was conducted in a urology outpatient setting, the patient population reflects individuals referred for or presenting with suspected urological conditions. Therefore, the findings should be interpreted within the context of this clinical setting.

### Inclusion and exclusion criteria

Female patients aged between 18 and 65 years who presented with newly developed flank pain during the study period were considered eligible for inclusion. Only patients with available laboratory and radiological imaging data obtained at the time of initial evaluation were included in the analysis to ensure consistency in diagnostic assessment. Patients were excluded if they had a previously diagnosed chronic urological disease, were experiencing active menstrual bleeding at the time of presentation, had incomplete medical records, or were outside the defined age range. The requirement for both laboratory and imaging data at presentation was applied to maintain a uniform diagnostic approach across the study population.

### Definition of study groups

Patients were categorized into two groups based on the presence or absence of a confirmed urological diagnosis established during the initial clinical evaluation.

Group 1 consisted of patients with a confirmed urological diagnosis, defined on the basis of compatible clinical findings supported by laboratory results and/or imaging studies. These diagnoses included conditions such as urinary tract infection, urolithiasis, hydronephrosis, and other urological pathologies documented in the medical records.

Group 2 included patients in whom no definitive urological diagnosis was established at the time of evaluation. This group represents patients without a confirmed urological etiology and does not necessarily correspond to patients with confirmed non-urological diagnoses.

Final diagnostic categorization, including the classification of specific urological conditions, was based on physician-documented diagnoses recorded in the electronic medical records.

### Clinical and diagnostic assessment

All patients underwent a structured clinical evaluation at the time of presentation. Clinical data included pain characteristics such as onset (acute or chronic), duration, laterality (right, left, or bilateral), and pain type (colicky, dull, or sharp). Associated symptoms, including fever, dysuria, nausea, vomiting, and hematuria, were systematically recorded. Given the broad differential diagnosis of flank pain in female patients, the clinical assessment also included evaluation for potential non-urological causes based on available clinical findings and medical records. These included musculoskeletal conditions, gastrointestinal disorders, and gynecological pathologies when clinically suspected. In addition, dermatological causes such as herpes zoster were considered during physical examination, particularly in patients presenting with localized pain or skin findings. Clinical decision-making was based on routine outpatient evaluation protocols, and diagnostic categorization was performed according to the findings documented in patient records [14].

### Laboratory assessments

Laboratory evaluations were performed at the time of presentation as part of routine clinical assessment. Serum creatinine levels were measured to evaluate renal function. Complete blood count parameters, including white blood cell (WBC) count, were recorded as indicators of systemic inflammatory response. Urinalysis was performed in all patients to assess urinary red blood cells (RBCs) and white blood cells (WBCs), which were considered markers of hematuria and urinary tract inflammation, respectively. Urinary WBC levels were used as indicators of potential infection or inflammatory processes affecting the urinary tract. In patients with clinical suspicion of urinary tract infection, urine culture results were reviewed when available and used to support diagnostic classification.

### Radiological assessments

Radiological imaging was utilized to identify structural abnormalities of the urinary tract as part of routine clinical evaluation. Ultrasonography (USG) was employed as the first-line imaging modality to assess for hydronephrosis, renal masses, and the presence of larger urinary stones. Non-contrast computed tomography of the kidneys, ureters, and bladder (CT-KUB) was performed when clinically indicated to confirm the presence, size, and location of urinary stones and to further evaluate equivocal findings on ultrasonography. The selection of imaging modality was based on clinical judgment and routine diagnostic protocols of the outpatient clinic. Imaging findings were used to support clinical diagnosis but were not included as independent variables in multivariable regression analyses to avoid potential circularity with the outcome definition.

### Statistical analysis

All statistical analyses were performed using IBM SPSS Statistics for Windows (version 26.0; IBM Corp., Armonk, NY, USA). Continuous variables were assessed for normality using the Kolmogorov–Smirnov test in conjunction with visual methods, including histograms and Q–Q plots. Normally distributed variables are presented as mean ± standard deviation, whereas non-normally distributed variables are presented as median (interquartile range). Comparisons between groups were conducted using the independent samples t-test for normally distributed variables and the Mann–Whitney U test for non-normally distributed variables. Categorical variables are expressed as frequencies and percentages and were compared using the chi-square test. Receiver operating characteristic (ROC) curve analysis was performed to evaluate the diagnostic performance of laboratory parameters. The area under the curve (AUC) was calculated, and optimal cutoff values were determined using the Youden index. In addition, combined diagnostic models incorporating laboratory and clinical variables were evaluated. Specifically, combinations of urinary WBC with serum creatinine and clinical symptoms (e.g., colicky pain) were assessed to determine their discriminative performance. Univariable analyses were initially performed to identify variables associated with a confirmed urological diagnosis. Variables with a p-value < 0.05 in univariable analyses were subsequently included in the multivariable logistic regression model. Among the candidate variables, only laboratory parameters met the inclusion criteria for the final multivariable model. To avoid circularity between predictors and outcome definitions, imaging-derived variables were not included in the multivariable analysis. Results of the regression analyses are presented as odds ratios (ORs) with corresponding 95% confidence intervals (CIs). A two-sided p-value < 0.05 was considered statistically significant.

## Results

A total of 3,350 female patients presenting with flank pain were included in the analysis. Of these, 1,831 patients (54.7%) were classified as having a confirmed urological diagnosis (Group 1), whereas 1,519 patients (45.3%) had no confirmed urological diagnosis (Group 2). As shown in Table 1, the mean age of the study population was comparable between the two groups (44.6 ± 12.9 years in Group 1 vs. 44.3 ± 12.9 years in Group 2, *p* = 0.433), indicating no statistically significant difference between groups.

**Table 1:** Baseline demographic and laboratory characteristics of the study population

Parameter	Group 1 (Confirmed Urological Diagnosis, *n* = 1831)	Group 2 (No Confirmed Urological Diagnosis, *n* = 1519)	*p*-value
Age (years)	44.6 ± 12.9	44.3 ± 12.9	0.433
BMI (kg/m²)	26.4 ± 4.2	26.1 ± 4.0	0.321
Hypertension, n (%)	588 (32.1%)	463 (30.5%)	0.048*
Diabetes mellitus, n (%)	342 (18.7%)	261 (17.2%)	0.039*
Serum creatinine (mg/dL)	0.89 ± 0.80	0.79 ± 0.55	0.02*
White blood cell count (×10³/mm³)	8.19 ± 2.84	7.81 ± 2.80	0.01*
Urinary RBCs (/HPF)	4.31 ± 0.52	4.36 ± 0.44	0.110
Urinary WBCs (/HPF)	92.3 ± 398.9	1.17 ± 1.47	0.01*

*BMI* Body mass index, *WBC* White blood cell, *RBC* Red blood cell, *HPF* High-power field

Continuous variables are presented as mean ± standard deviation and categorical variables as n (%)

*Statistically significant values are indicated with an asterisk (*p* < 0.05)

Imaging findings of the study population are presented in Table 2. Hydronephrosis was identified in 278 patients (15.2%) in Group 1 and in 77 patients (5.1%) in Group 2 (*p* = 0.01). Urinary stones were detected in 362 patients (19.8%) in Group 1 and in 20 patients (1.3%) in Group 2 (*p* = 0.01).

**Table 2:** Imaging findings of the study population

Imaging Findings	Group 1 (Confirmed Urological Diagnosis, *n* = 1831)	Group 2 (No Confirmed Urological Diagnosis, *n* = 1519)	*p*-value
Hydronephrosis on imaging, n (%)	278 (15.2%)	77 (5.1%)	0.01*
Presence of urinary stones, n (%)	362 (19.8%)	20 (1.3%)	0.01*

Categorical variables are presented as n (%)

*Statistically significant values are indicated with an asterisk (*p* < 0.05)

The diagnostic performance of laboratory parameters for identifying patients with a confirmed urological diagnosis is presented in Table 3. Receiver operating characteristic (ROC) curve analysis showed that urinary WBC levels had an area under the curve (AUC) of 0.82 (95% CI: 0.78–0.86, *p* = 0.01), whereas serum creatinine had an AUC of 0.76 (95% CI: 0.72–0.80, *p* = 0.02). The optimal cutoff value for urinary WBC was 40/HPF, with a sensitivity of 83.7% and a specificity of 76.1%. For serum creatinine, the optimal cutoff value was 0.86 mg/dL, yielding a sensitivity of 79.5% and a specificity of 68.2%.

**Table 3:** Receiver Operating Characteristic (ROC) analysis of laboratory parameters

Parameter	AUC (95% CI)	Optimal Cutoff	Sensitivity (%)	Specificity (%)	*p*-value
Serum creatinine	0.76 (0.72–0.80)	0.86 mg/dL	79.5%	68.2%	0.02*
Urinary WBCs	0.82 (0.78–0.86)	40/HPF	83.7%	76.1%	0.01*

*AUC* Area under the curve, *CI* Confidence interval, *HPF* High-power field, *WBC* White blood cell

Diagnostic performance was assessed using ROC curve analysis; optimal cutoffs were determined by the Youden index

*Statistically significant values are indicated with an asterisk (*p* < 0.05)

A multivariable logistic regression analysis was performed to identify factors associated with a confirmed urological diagnosis. Variables that were statistically significant in univariable analyses were entered into the multivariable model. To avoid potential circularity, imaging-derived variables were not included in the model. As shown in Table 4, in the adjusted analysis, urinary WBC levels and serum creatinine remained significantly associated with a confirmed urological diagnosis. Elevated urinary WBC levels were associated with increased odds of a confirmed urological diagnosis (OR: 3.12, 95% CI: 2.64–3.68, *p* = 0.01), while higher serum creatinine levels were also independently associated (OR: 2.08, 95% CI: 1.72–2.51, *p* = 0.03).

**Table 4:** Multivariable logistic regression analysis for factors associated with confirmed urological diagnosis

Variable	Odds Ratio (95% CI)	*p*-value
Serum creatinine	2.08 (1.72–2.51)	0.03*
Urinary WBCs	3.12 (2.64–3.68)	0.01*

*OR* Odds ratio, *CI* Confidence interval, *WBC* White blood cell

The model included variables with *p* < 0.05 in univariable analyses; imaging variables were excluded to avoid circularity

*Statistically significant values are indicated with an asterisk (*p* < 0.05)

The distribution of clinical symptoms according to diagnostic groups is presented in Table 5. Acute onset pain was reported in 1124 patients (61.4%) in Group 1 and in 427 patients (28.1%) in Group 2 (*p* = 0.02). Colicky pain was observed in 1079 patients (58.9%) in Group 1 compared with 292 patients (19.2%) in Group 2 (*p* = 0.01). Dysuria was present in 782 patients (42.7%) in Group 1 and in 188 patients (12.4%) in Group 2 (*p* = 0.05).

**Table 5:** Distribution of clinical symptoms according to diagnostic groups

Symptom	Group 1 (Confirmed Urological Diagnosis, *n* = 1831)	Group 2 (No Confirmed Urological Diagnosis, *n* = 1519)	*p*-value
Acute pain, n (%)	1124 (61.4%)	427 (28.1%)	0.02*
Colicky pain, n (%)	1079 (58.9%)	292 (19.2%)	0.01*
Dysuria, n (%)	782 (42.7%)	188 (12.4%)	0.05

Categorical variables are presented as n (%)

*Statistically significant values are indicated with an asterisk (*p* < 0.05)

The diagnostic performance of combined clinical and laboratory parameters is presented in Table 6. Urinary WBC levels alone showed an AUC of 0.82 (95% CI: 0.78–0.86, *p* = 0.01), while serum creatinine alone had an AUC of 0.76 (95% CI: 0.72–0.80, *p* = 0.02). The combination of urinary WBC and serum creatinine yielded an AUC of 0.84 (95% CI: 0.80–0.88, *p* = 0.01), with a sensitivity of 85.2% and a specificity of 78.4%. The combination of urinary WBC and colicky pain demonstrated an AUC of 0.86 (95% CI: 0.82–0.89, *p* = 0.01), with a sensitivity of 87.1% and a specificity of 80.3%.

**Table 6:** Diagnostic performance of combined clinical and laboratory parameters

Parameter Combination	AUC (95% CI)	Sensitivity (%)	Specificity (%)	*p*-value
Urinary WBC alone	0.82 (0.78–0.86)	83.7%	76.1%	0.01*
Serum creatinine alone	0.76 (0.72–0.80)	79.5%	68.2%	0.02*
Urinary WBC + Serum creatinine	0.84 (0.80–0.88)	85.2%	78.4%	0.01*
Urinary WBC + Colicky pain	0.86 (0.82–0.89)	87.1%	80.3%	0.01*

*AUC* Area under the curve, *CI* Confidence interval, *WBC* White blood cell

Diagnostic performance was assessed using ROC curve analysis; optimal thresholds were determined using the Youden index

*Statistically significant values are indicated with an asterisk (*p* < 0.05)

The distribution characteristics of key laboratory parameters are presented in Table 7. Urinary WBC values demonstrated a wide dispersion in both groups, with higher mean and median values observed in patients with a confirmed urological diagnosis. Median (interquartile range) values for urinary WBCs were 38 (12–95) /HPF in Group 1 and 1 (0–2) /HPF in Group 2 (*p* = 0.01).

**Table 7:** Distribution characteristics of laboratory parameters

Parameter	Group 1 (Confirmed Urological Diagnosis, *n* = 1831)	Group 2 (No Confirmed Urological Diagnosis, *n* = 1519)	*p*-value
Urinary WBCs (/HPF), mean ± SD	92.3 ± 398.9	1.17 ± 1.47	0.01*
Urinary WBCs (/HPF), median (IQR)	38 (12–95)	1 (0–2)	0.01*
Serum creatinine (mg/dL), mean ± SD	0.89 ± 0.80	0.79 ± 0.55	0.02*
Urinary RBCs (/HPF), mean ± SD	4.31 ± 0.52	4.36 ± 0.44	0.110

*SD* Standard deviation, *IQR* Interquartile range, *HPF* High-power field, *WBC* White blood cell, *RBC* Red blood cell

Continuous variables are presented as mean ± SD or median (IQR), as appropriate. Normally distributed variables were compared using the independent samples t-test, whereas non-normally distributed variables were compared using the Mann–Whitney U test

*Statistically significant values are indicated with an asterisk (*p* < 0.05)

Serum creatinine levels were 0.89 ± 0.80 mg/dL in Group 1 and 0.79 ± 0.55 mg/dL in Group 2 (*p* = 0.02). Urinary RBC values were similar between the two groups (4.31 ± 0.52 vs. 4.36 ± 0.44 /HPF, *p* = 0.110).

Subgroup analyses were performed among patients with a confirmed urological diagnosis (Group 1) to describe the distribution of specific etiologies. Urinary tract infection and urolithiasis were the most frequently identified diagnoses. Other urological conditions were recorded as documented in the medical records. The distribution of urological diagnoses is presented in Table 8.

**Table 8:** Distribution of urological diagnoses in patients with confirmed urological pathology (Group 1)

Diagnosis	*n* (%)
Urinary tract infection	509 (27.8%)
Urolithiasis	362 (19.8%)
Hydronephrosis without visible stone	214 (11.7%)
Acute pyelonephritis	186 (10.2%)
Renal cyst	148 (8.1%)
Ureteral stricture/obstruction	132 (7.2%)
Other urological conditions*	280 (15.3%)
**Total**	**1831 (100%)**

Values are presented as n (%). Other urological conditions include less frequently encountered diagnoses recorded in patient files

## Discussion

This study aimed to evaluate the etiology of flank pain in female patients and assess the diagnostic value of laboratory markers and imaging findings in differentiating between urological and non-urological causes. Rather than solely describing etiological distribution, the present study additionally evaluated the combined diagnostic performance of clinical, laboratory, and imaging parameters within a real-world urology outpatient setting. This approach provides a more clinically applicable framework for diagnostic decision-making by integrating routinely available parameters into a structured evaluation process. Our results demonstrated that while serum creatinine and urinary WBC counts were significantly higher in patients with urological flank pain, these markers alone were insufficient for definitive diagnosis. Imaging findings play a crucial role in confirming the diagnosis, highlighting the importance of an integrated diagnostic approach that combines clinical history, laboratory parameters, and imaging modalities.

Flank pain is a frequent presenting complaint in both emergency and outpatient settings, with significant clinical and healthcare burdens [15, 16]. In this study, 54.7% of the patients had a confirmed urological diagnosis, whereas 45.3% had no confirmed urological etiology at initial evaluation. These findings are consistent with previous reports, including the study by Kim et al., which demonstrated that approximately 55% of patients presenting with flank pain had a urological pathology [17]. Among urological conditions, urinary tract infections (27.8%) and urolithiasis (19.8%) were the most frequently observed. However, these findings should be interpreted in the context of a urology outpatient setting, where patients are more likely to be referred with suspected urological conditions, potentially leading to an overrepresentation of urological diagnoses.

Differentiating between urological and non-urological causes remains a clinical challenge, as symptom overlap frequently leads to diagnostic uncertainty [18]. While urinary tract infections and urolithiasis typically present with localized, colicky pain and urinary symptoms, musculoskeletal and gastrointestinal disorders may mimic these presentations, increasing the risk of misdiagnosis and unnecessary interventions. Importantly, this study extends beyond descriptive epidemiology by evaluating the combined diagnostic performance of routinely used clinical and laboratory parameters, providing a more practical and clinically relevant framework for diagnostic decision-making [19, 20].

Among non-urological causes, musculoskeletal disorders (25.5%) and gastrointestinal conditions (14.7%) were the most frequently identified. However, non-urological diagnoses were not systematically confirmed in all patients, and Group 2 represents the absence of a confirmed urological diagnosis rather than a fully characterized non-urological cohort. Fibromyalgia, myofascial pain syndrome, and spinal disorders are well-recognized causes of chronic flank pain and may be misclassified as urological conditions [21, 22]. These findings highlight the importance of a multidisciplinary approach, particularly in patients with persistent or non-specific symptoms.

The higher prevalence of hypertension and diabetes mellitus in patients with confirmed urological diagnoses may be explained by underlying metabolic and microvascular mechanisms. Chronic vascular changes, immune dysregulation, and increased susceptibility to infection in these populations may contribute to both renal dysfunction and increased risk of urinary tract infections. These findings suggest that metabolic comorbidities should be considered during the clinical evaluation of patients presenting with flank pain.

Serum creatinine is widely used as a marker of renal function; however, its diagnostic value in differentiating flank pain etiologies remains limited. Although serum creatinine levels were significantly higher in the urological group (*p* = 0.02), ROC analysis demonstrated only moderate diagnostic performance (AUC: 0.76). These findings are consistent with previous studies indicating that serum creatinine alone lacks sufficient sensitivity and specificity for diagnosing urological pathology [23–25]. Therefore, it should be interpreted in conjunction with other clinical and laboratory findings.

Given the limitations of individual biomarkers, composite diagnostic models incorporating inflammatory markers such as C-reactive protein, neutrophil-to-lymphocyte ratio, or procalcitonin have been proposed [26, 27]. In line with this approach, our findings demonstrated that combined models incorporating laboratory and clinical parameters showed improved discriminative performance compared to individual markers alone.

Urinary WBC counts were significantly elevated in the urological group (*p* = 0.01), with an AUC of 0.82, supporting their role in identifying infectious and obstructive urological conditions. However, their specificity was moderate, indicating that elevated values may also occur in non-urological conditions. Therefore, urinary WBC should not be used in isolation but rather as part of a multiparametric diagnostic approach [28].

Imaging plays a pivotal role in distinguishing urological from non-urological causes of flank pain [29]. Hydronephrosis and urolithiasis were significantly more common in the urological group (*p* = 0.01), underscoring their diagnostic importance. Non-contrast computed tomography (CT-KUB) remains the gold standard for detecting urinary stones, with high sensitivity and specificity. However, imaging variables were not included in multivariable analyses in this study to avoid circularity between diagnostic criteria and outcome definition.

Excessive reliance on imaging may lead to unnecessary radiation exposure. In patients with low clinical suspicion, ultrasonography may serve as an appropriate initial modality, particularly in younger or pregnant patients. Future research should aim to refine imaging strategies and incorporate risk-based approaches.

Pain characteristics also provide valuable diagnostic clues. Colicky pain was significantly associated with urological causes (*p* = 0.01), whereas chronic pain was more commonly observed in non-urological conditions [30]. These findings emphasize the importance of detailed clinical assessment in guiding diagnostic pathways.

Although no significant difference in BMI was observed between groups, obesity remains a well-established risk factor for urolithiasis and urinary tract infections. Metabolic alterations such as insulin resistance, hyperuricosuria, and chronic low-grade inflammation may contribute to disease development. The absence of a significant association in this study may be related to incomplete BMI data and the retrospective design.

This study provides valuable clinical insights due to its large sample size and comprehensive evaluation approach. However, several limitations must be acknowledged. The retrospective design limits standardization and introduces potential selection and information bias. The inclusion of only patients who underwent both laboratory and imaging evaluations may have introduced work-up bias, potentially limiting generalizability.

Group 2 was defined as patients without a confirmed urological diagnosis, which may include undiagnosed urological conditions or incomplete follow-up, introducing potential misclassification bias. Additionally, body mass index and microbiological data were not consistently available, limiting further subgroup analyses. Imaging findings were also restricted to certain modalities, and comparative analyses were not performed.

The single-center urology outpatient setting may further limit external validity and contribute to an overrepresentation of urological diagnoses. In addition, the lack of standardized follow-up limited confirmation of final diagnoses in all patients. Residual confounding cannot be excluded due to incomplete data on certain clinical variables.

Future studies should focus on prospective, multicenter designs incorporating broader biomarkers and advanced imaging techniques. The integration of artificial intelligence-based decision support systems may further enhance diagnostic accuracy and clinical workflow efficiency.

## Conclusions

This study demonstrated that urological causes account for a substantial proportion of flank pain presentations in female patients evaluated in a urology outpatient setting, with urinary tract infections and urolithiasis being the most frequently documented conditions. While urinary WBC counts, serum creatinine measurements, and imaging findings provide useful diagnostic information, no single parameter alone is sufficient for definitive diagnosis. These findings support the need for a structured, multiparametric approach integrating clinical assessment, laboratory markers, and imaging to improve diagnostic accuracy.

Pain characteristics also play an important role in the initial evaluation of flank pain. Factors such as onset, severity, laterality, and associated symptoms should be assessed together with laboratory and radiological findings. A structured diagnostic workflow may help reduce unnecessary testing and imaging, particularly in patients with low clinical suspicion of urological pathology.

The findings highlight the importance of refining diagnostic strategies for flank pain in female patients with a broad differential diagnosis. Future studies should explore the role of novel biomarkers, advanced imaging, and artificial intelligence–based decision support systems. Nevertheless, the results should be interpreted in light of the retrospective design and specific clinical setting.

## Data Availability

The data that support the findings of this study are available from the corresponding author upon reasonable request. Requests for access to the data should be directed to the corresponding author via email.

## Abbreviations

AUC: Area under the curve
BMI: Body mass index
CI: Confidence interval
CT-KUB: Computed Tomography of the Kidneys, Ureters, and Bladder
HPF: Highpower field
OR: Odds ratio
RBC: Red blood cells
USG: Ultrasonography
WBC: White blood cell
